# RETRACTED: Kelly et al. Delineating Conformational Variability in Small Protein Structures Using Combinatorial Refinement Strategies. *Micromachines* 2023, *14*, 1869

**DOI:** 10.3390/mi16121317

**Published:** 2025-11-25

**Authors:** Deborah F. Kelly, G M Jonaid, Liam Kaylor, Maria J. Solares, Samantha Berry, Liza-Anastasia DiCecco, William Dearnaley, Michael Casasanta

**Affiliations:** 1 Department of Biomedical Engineering, Pennsylvania State University, University Park, PA 16802, USA; 2 Center for Structural Oncology, Pennsylvania State University, University Park, PA 16802, USA; 3 Bioinformatics and Genomics Graduate Program, Huck Institutes of the Life Sciences, Pennsylvania State University, University Park, PA 16802, USA; 4 Molecular, Cellular, and Integrative Biosciences Graduate Program, Huck Institutes of the Life Sciences, Pennsylvania State University, University Park, PA 16802, USA

The Journal retracts the article titled “Delineating Conformational Variability in Small Protein Structures Using Combinatorial Refinement Strategies” [1], cited above.

Following the publication, concerns were brought to the attention of the Editorial Office about the reliability of the electron microscopy data presented in this paper [1].

An investigation was conducted by Penn State University that confirmed that the dataset presented in this study did not meet the acceptable standards and norms. In addition, dimer and monomer maps show no secondary structure nor domain features that would allow rational modelling. The Editorial Office and Editorial Board evaluated the investigation by the above-mentioned institution and concurred with the presented findings and recommendation. Consequently, the Editorial Office and Editorial Board have decided to retract, as per MDPI’s retraction policy (https://www.mdpi.com/ethics#_bookmark30).

This retraction was approved by the Section Editor-in-Chief of the journal *Micromachines*.

The authors did not provide a comment on this decision.
